# Genes encoding SATB2-interacting proteins in adult cerebral cortex contribute to human cognitive ability

**DOI:** 10.1371/journal.pgen.1007890

**Published:** 2019-02-06

**Authors:** Isabella Cera, Laura Whitton, Gary Donohoe, Derek W. Morris, Georg Dechant, Galina Apostolova

**Affiliations:** 1 Institute for Neuroscience, Medical University of Innsbruck, Innsbruck, Austria; 2 Cognitive Genetics and Cognitive Therapy Group, Neuroimaging, Cognition and Genomics (NICOG) Centre and NCBES Galway Neuroscience Centre, School of Psychology and Discipline of Biochemistry, National University of Ireland Galway, Galway, Ireland; Cardiff University, UNITED KINGDOM

## Abstract

During CNS development, the nuclear protein SATB2 is expressed in superficial cortical layers and determines projection neuron identity. In the adult CNS, SATB2 is expressed in pyramidal neurons of all cortical layers and is a regulator of synaptic plasticity and long-term memory. Common variation in *SATB2* locus confers risk of schizophrenia, whereas rare, *de novo* structural and single nucleotide variants cause severe intellectual disability and absent or limited speech. To characterize differences in SATB2 molecular function in developing vs adult neocortex, we isolated SATB2 protein interactomes at the two ontogenetic stages and identified multiple novel SATB2 interactors. SATB2 interactomes are highly enriched for proteins that stabilize *de novo* chromatin loops. The comparison between the neonatal and adult SATB2 protein complexes indicates a developmental shift in SATB2 molecular function, from transcriptional repression towards organization of chromosomal superstructure. Accordingly, gene sets regulated by SATB2 in the neocortex of neonatal and adult mice show limited overlap. Genes encoding SATB2 protein interactors were grouped for gene set analysis of human GWAS data. Common variants associated with human cognitive ability are enriched within the genes encoding adult but not neonatal SATB2 interactors. Our data support a shift in the function of SATB2 in cortex over lifetime and indicate that regulation of spatial chromatin architecture by the SATB2 interactome contributes to cognitive function in the general population.

## Author summary

SATB2 is a homeodomain protein that recruits transcriptional and epigenetic regulators to its DNA binding sites. We have recently shown that deletion of *Satb2* from the forebrain of adult mice leads to impaired long-term memory. Human patients with *SATB2* haploinsufficiency suffer from severe neurological symptoms including cognitive deficits, developmental delay and absent/limited speech. Here, we tested the hypothesis that SATB2 molecular functions differ between developing and adult cortex. Our results provide experimental support for this concept by demonstrating that 1) SATB2 interacts with different protein networks at the two ontogenetic stages, with a switch from transcriptional repression towards organization of chromatin structure and 2) SATB2 determines differential transcriptional programs in neonatal vs adult cortex. To explore the contribution of SATB2 interactomes to human cognition, we tested the sets of genes encoding SATB2 interactors for enrichment using the largest available genome-wide association studies (GWAS) datasets for cognitive ability and neuropsychiatric diseases. We found that genes encoding SATB2 interactomes are highly constrained. Rare high impact mutations in these genes cause severe cognitive disorders whereas common low impact variants influence general cognitive ability in the population. Given that SATB2 is a known 3D-genome organizer protein, our data emphasize the role of long-range chromatin interactions in human cognition and present novel and unsuspected candidates for further studies on the underpinnings of intelligence and the mechanisms underlying intelligence differences.

## Introduction

SATB2 is a highly conserved nuclear protein that binds to matrix attachment regions in DNA via a homeodomain and two CUT domains [1]. In the embryonic cortex, SATB2 is expressed in superficial cortical layers and determines callosal vs. subcortical projection neuron identity by repressing *Ctip2* transcription [2–4]. During CNS maturation SATB2 expression shifts towards the deep cortical layers and in the adult brain SATB2 is expressed in pyramidal neurons of all layers of the cerebral cortex and in the CA1 area of the hippocampus [5], indicating a function in cognition. By eliminating SATB2 selectively from the mouse forebrain after the 3^rd^ postnatal week of life, we and others have shown that it is indeed required for stabilization of long-term potentiation and long-term memory in the adult CNS [6,7].

In humans patients, alterations of the *SATB2* gene (*de novo* structural or single nucleotide variants) cause developmental delay, intellectual disability, limited to absent speech and behavioral issues [8–14]. Human *SATB2* has also been identified as a risk locus for schizophrenia [15,16]. To which extent symptoms in SATB2-related human pathologies depend on developmental or adult functions of the protein remains to be established.

Potential approaches to address differential function of a transcriptional regulator include defining its protein interactors and characterizing the transcriptional responses that depend on it. SATB2-driven gene expression programs in the neonatal mouse cortex have previously been identified [17]. At protein level, *in vitro* and *in vivo* assays have implicated interactions of SATB2 in the embryonic cortex with components of the NuRD complex, including histone deacetylase 1 (HDAC1) and metastasis-associated protein 2 (MTA2), as well as with the SKI protein causing repression of the *Ctip2* locus and active suppression of the subcortical projection neuron fate [3,18]. However, SATB2 multiprotein complexes have not been analyzed using unbiased proteomic approaches, either in the developing or the adult cortex. Also unknown are SATB2-driven changes in gene transcription in the adult cortex. Here, we combine proteomic and transcriptomic approaches to characterize and compare SATB2 interacting partners and SATB2 transcriptional activity in neonatal vs. adult mouse cortex. We show that SATB2 interacts with different protein networks at the two ontogenetic stages and regulates distinct gene expression programs linked to cell projection morphogenesis and brain development at the neonatal stage vs synapse, neurotransmitter transport, and calcium ion binding and signaling at the adult stage. By combining our unbiased proteomic findings with analyses of human genetic data, we demonstrate that the genes encoding adult but not those coding for neonatal SATB2 interactors are enriched in common variants associated with cognitive function in the general population.

## Results

### Proteomic analysis of neuronal SATB2 complexes uncovers novel specific interactors

**Fig 1:**
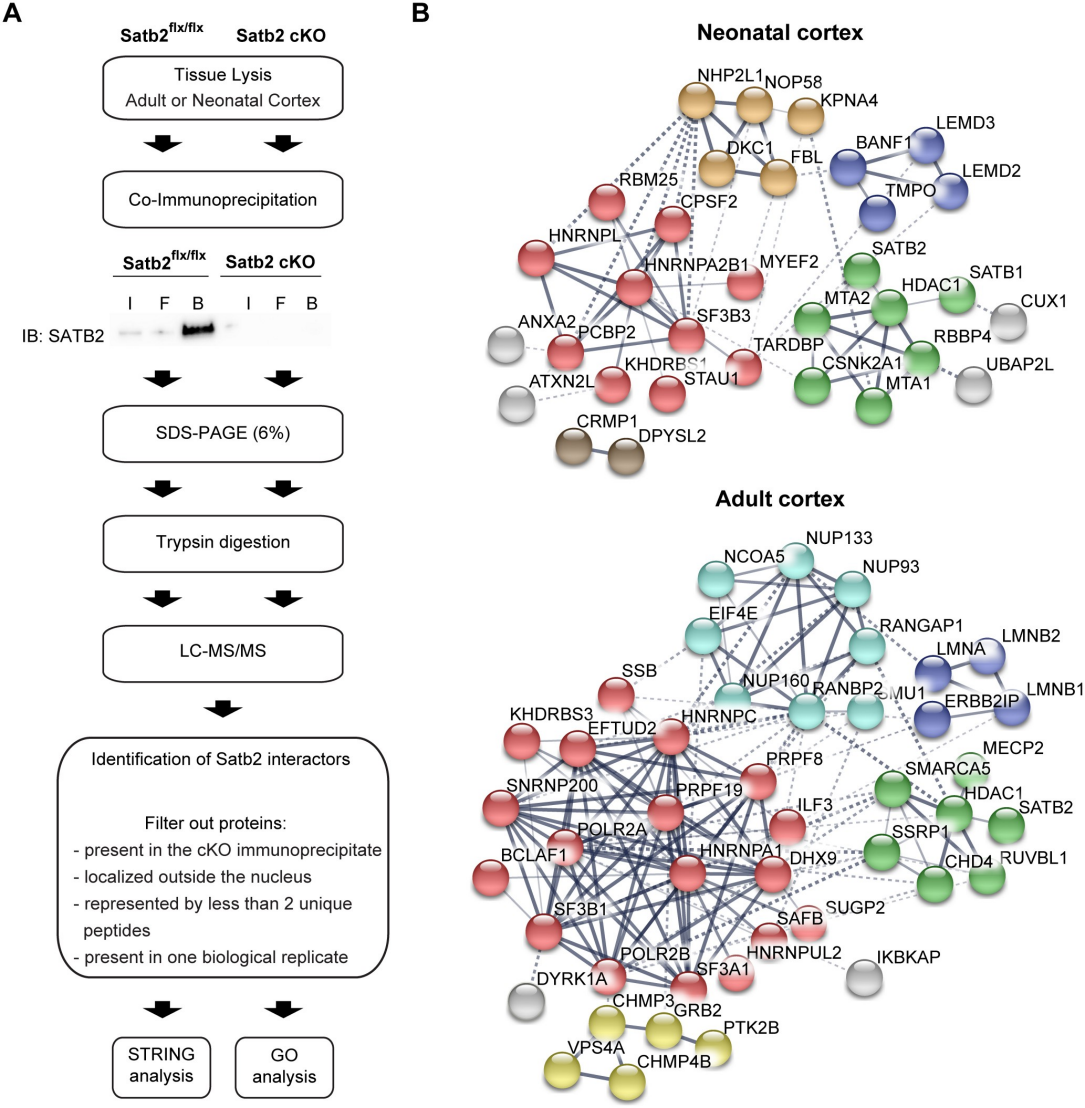
Proteomic analysis of SATB2 complexes in neonatal and adult mouse cortex. (A) Schematic of experimental workflow. IP were performed using cortical lysates from control (*Satb2*^*flx/flx*^) and *Satb2* cKO mice (adult cortex: *Satb2*^*flx/flx*^::*CamK2a-Cre*; neonatal cortex: *Satb2*^*flx/flx*^::*Nes-Cre*). (B) Protein-protein interaction networks of neonatal and adult cortex SATB2 interactomes, extracted from the STRING 10.5 database. Shown are only interactors that are connected within a network. Line thickness indicates the strength of data support. Clusters identified by k-means clustering are shown in different colors: red, RNA binding/processing proteins; green, HDAC1/NURD complex-associated proteins; dark blue, nuclear lamina-associated proteins; light blue, nuclear pore complex; yellow, ESCRTIII complex-associated proteins; orange, nucleolar proteins; brown, CRMP1- DPYSL2 cluster; grey, proteins without clearly defined association.

To identify SATB2-containing protein complexes, we immunoprecipitated endogenous SATB2 from neonatal and adult mouse cortical tissue and subjected the precipitates to liquid chromatography / mass spectrometry (MS) analysis (Fig 1A). Cortical lysate from *Satb2* knock out mice served as a negative control at both developmental stages. Additionally, we filtered out all non-nuclear proteins (6 in the neonatal cortex and 16 in the adult cortex SATB2 proteomes) by bioinformatics means. This analysis identified 40 proteins in the SATB2 immunoprecipitates from neonatal cortex (S1 Table) and 53 proteins in the SATB2 immunoprecipitates from adult cortex (S2 Table). The unfiltered neonatal and adult cortex SATB2 interactomes are provided in S8 and S9 Tables. Of note, the filtration based upon nuclear localization had minimal impact on the composition of the identified SATB2 proteomes (S1 Fig, S8 and S9 Tables), most likely due to the specificity already achieved by using a knock-out lysate as a negative control.

Among the neonatal SATB2 interactors were the previously reported HDAC1 and MTA family members [18–20]. All 90 remaining proteins represent newly identified SATB2 binding partners (Fig 1B). In line with the reported binding of SATB2 to nuclear matrix attachment regions of the DNA [19], we identified nuclear matrix proteins among the novel SATB2 interactors [21], including HNRNPs, casein kinase II (CKII), nucleolin, centromere binding protein (CNP) and scaffold attachment factor B1 (SAFB1).

**Fig 2:**
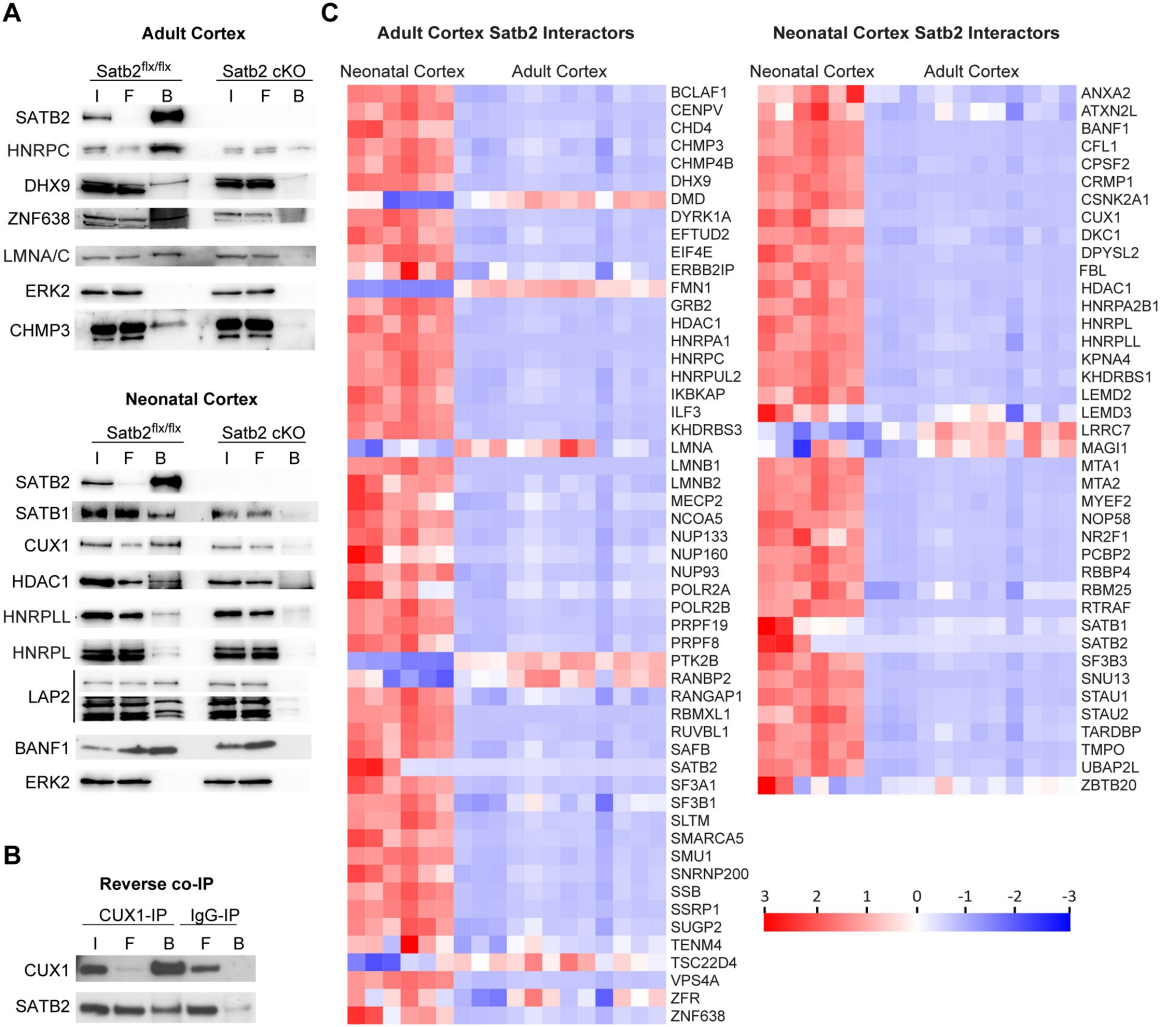
Validation of proteomics data. (A) Candidate SATB2 interacting proteins were strongly enriched in immunoprecipitates from cortical lysates of control but not SATB2-deficient mice by IB using the indicated antibodies. The equal input of total protein from control and SATB2-deficient cortical lysates was controlled by ERK2 detection. Representative images of the immunoblots are shown; I (Input); F (Flow-through); B (Beads). (B) Reverse IP with CUX1 and control IgG antibody, followed by IB using CUX1 and SATB2 antibodies. (C) Expression of SATB2 interactors in neonatal and adult mouse cortex. Heatmaps compare the expression levels (gene counts) of adult cortex SATB2 interactors (left) and neonatal cortex SATB2 interactors (right) in P0 vs adult mouse cortex. RNA-seq data from McKenna et al. [17] (P0 mouse cortex) and this study (adult mouse cortex) were used to generate the heatmaps by using FunRich Tool (http://www.funrich.org/). The color scale bar shows z-score values after z-score row normalization.

To validate MS data, we performed independent direct and reverse immunoprecipitations (IP) from cortical lysates, followed by immunoblotting (IB) with specific antibodies. Heterogeneous nuclear ribonucleoprotein L (HNRNPL), HNRNPL-like, HNRNPC, and DHX9 were chosen for validation from the group of RNA-binding proteins; CUX1, SATB1, and ZFN638 were selected as representatives of the transcription factor group; HDAC1 was included as a member of the NuRD complex [18,20]; Lamin A/C, BAF (barrier-to-autointegration factor) and LAP2—as representatives of the nuclear lamina. All tested novel SATB2-binding partners were highly enriched in precipitates from wild-type lysates compared to SATB2-deficient lysates (Fig 2A). SATB2 was also readily immunoprecipitated by a CUX1-directed antibody in a reciprocal IP, but not by a control rabbit IgG (Fig 2B).

To reveal functional relationships among the identified SATB2-interacting proteins, we used the STRING database [22]. Besides the NuRD complex [3,18,20], we identified several novel SATB2-containing protein complexes, such as nuclear lamina-associated protein complex (lamins, the LEM domain proteins LEM2, LEM3, LAP2, and BAF), nuclear pore complex, RNA-binding/processing proteins and nucleolar proteins (Fig 1B). The interactions of SATB2 with proteins of the nuclear pore complex were specific for the adult cortex, whereas the nucleolar proteins (NOP58, NHP2L1, DKC1, and FBL) were found exclusively in the neonatal interactome. The protein complexes containing RNA-binding/processing proteins, nuclear lamina-associated proteins, and HDAC1-associated proteins were shared between the neonatal and adult SATB2 interactomes, although individual protein components differed (Fig 1B). To test if this is determined by differences in expression levels of the interacting proteins, we compared the mRNA expression of SATB2-binding partners in neonatal vs. adult cortex (Fig 2C). We observed a decreased expression of almost all neonatal SATB2 binding proteins in the adult cortex compared to neonatal cortex, thus providing a potential explanation for the lack of detected interactions in the adult stage. Conversely, while some of the adult cortex SATB2 interactors (such as DMD, FMN1, LMNA, PTK2B, RANBP2, TSC22D4) showed increased expression in the adult compared to neonatal cortex, the majority of them had lower expression levels in the adult cortex, indicating that changes in expression are insufficient to explain the differences in the composition of the two interactomes.

**Fig 3:**
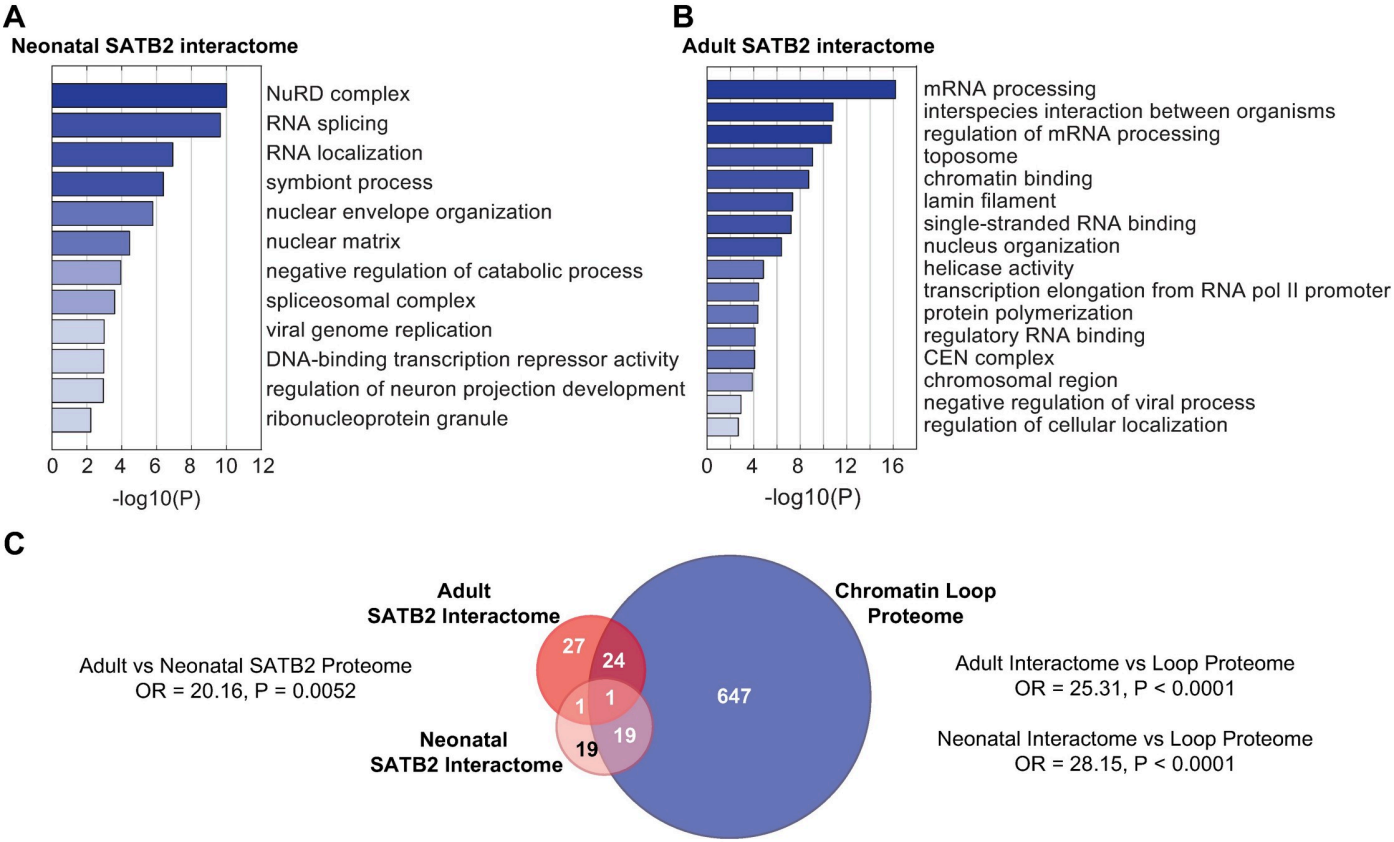
Functional enrichment analysis of SATB2 interactors. (A) Metascape enrichment analysis of neonatal cortex SATB2 interactome. (B) Metascape enrichment analysis of adult cortex SATB2 interactome. Shown are heatmaps of enriched terms across input protein lists, colored by p-values. (C) Venn diagram depicting the overlap between neonatal cortex SATB2 interactome, adult cortex SATB2 interactome, and proteins differentially enriched at stable chromatin loops [23] (Fischer’s exact test, adult interactome vs loop proteome, OR = 25.31, P < 0.0001; neonatal interactome vs loop proteome, OR = 28.15, P < 0.0001; neonatal vs adult proteome (including SATB2 itself), OR = 20.16, P = 0.0052; neonatal vs adult proteome (excluding SATB2 itself), OR = 10.08, P = 0.0984; background, 19 626 protein-coding genes). OR, odds ratio.

Next, we used the ConsensusPathDB bioinformatics tool to test which experimentally verified mammalian protein complexes are overrepresented in the neonatal and adult cortex SATB2 proteomes. Consistent with the previously described function of SATB2 as transcriptional repressor, the most enriched protein complexes in the neonatal SATB2 interactome were the SNF2h-cohesin-NuRD complex and the MTA1-HDAC core complex (S3 Table). In contrast, the most overrepresented protein complexes in the adult cortex SATB2 proteome were C-complex spliceosome, Toposome, ATAC B Complex, Lamin A/C/Lamin B1/Lamin B2, Spliceosome, capped, methylated pre-mRNP: CBC complex, and RanGAP/RanBP1/RanBP2 (S4 Table). In agreement with these observations, the gene ontology (GO) enrichment analysis by the Metascape tool also revealed different overrepresented biological pathways and GO terms in the neonatal vs adult SATB2 cortical interactomes (Fig 3A and 3B, S1 Fig). We found enrichment of NURD Complex, RNA splicing in the neonatal SATB2 interactome, in contrast to toposome, chromatin binding, nuclear organization found in the adult SATB2 intractome.

Taken together, these results indicate a shift in the functions of neonatal vs. adult cortex SATB2 interactome from transcriptional repression towards organization of chromosomal structure.

Of note, SATB2-binding partners in both neonatal and adult cortex were significantly enriched in proteins found at stable *de novo* chromatin loops [23] (Fig 2C) (adult SATB2 interactome, OR = 25.31, p < 0.0001; neonatal SATB2 interactome, OR = 28.15, p < 0.0001). Among the shared proteins were members of the HNRNP machinery (HNRNPL, HNRNPL-like, HNRNPC, HNRNPU-like 2, HNRNPA2/B1) and the ATP-dependent RNA helicase A (DHX9) known to be specifically recruited to long-term stable chromatin loops and required for stabilizing loop topology [23]. This finding is consistent with a function of SATB2 as a chromosomal scaffolding protein [24] and higher-order chromatin organizer [25].

### SATB2 determines differential transcriptional programs in neonatal vs adult cortex

Our proteomic analysis demonstrated differences in the composition of SATB2 protein complexes at neonatal vs adult developmental stage. To explore if these differences contribute to differential gene regulation by SATB2, we compared the sets of genes that are influenced by SATB2 in the cortex at the two ontogenetic stages. We performed gene expression analysis of adult cortex of SATB2-deficient and control mice by RNA-seq and identified 1157 differentially expressed genes (adjusted p value <0. 05). To allow for a correct comparison, we re-analyzed the RNA-seq data published by McKenna et al. [17] that describe transcriptome changes in P0 SATB2-mutant cortices. We applied identical bioinformatics procedures (pre-processing steps and threshold for differential expression) in the analysis of the two RNA-seq datasets. To compare the global gene expression signatures of SATB2 mutant vs wild-type cortex at neonatal and adult stage, we first applied a threshold-free approach using the rank-rank hypergeometric overlap (RRHO) analysis [26]. This algorithm ranks the entire gene lists (without introducing any cutoffs) according to a signed log_10_-transformed *t*-test *P*-value and steps through the two gene lists to calculate if the number of overlapping genes is significantly more or less than would be expected by chance. The RRHO heatmap (visualizing the matrix of the hypergeometric P-values) and the rank-rank scatter plot (in which each gene is plotted by its rank based on the direction-signed, log_10_-transformed *t*-test *P*-values in each gene list) demonstrated only a very weak overlap between the neonatal and adult cortex gene sets (Spearman’s ρ rank correlation coefficient = 0.013). The overlap is mostly in the genes up-regulated in the SATB2 mutant cortex (upper right quadrant in the RRHO heatmap), thus supporting a conserved function of SATB2 as transcriptional repressor at both developmental stages.

**Fig 4:**
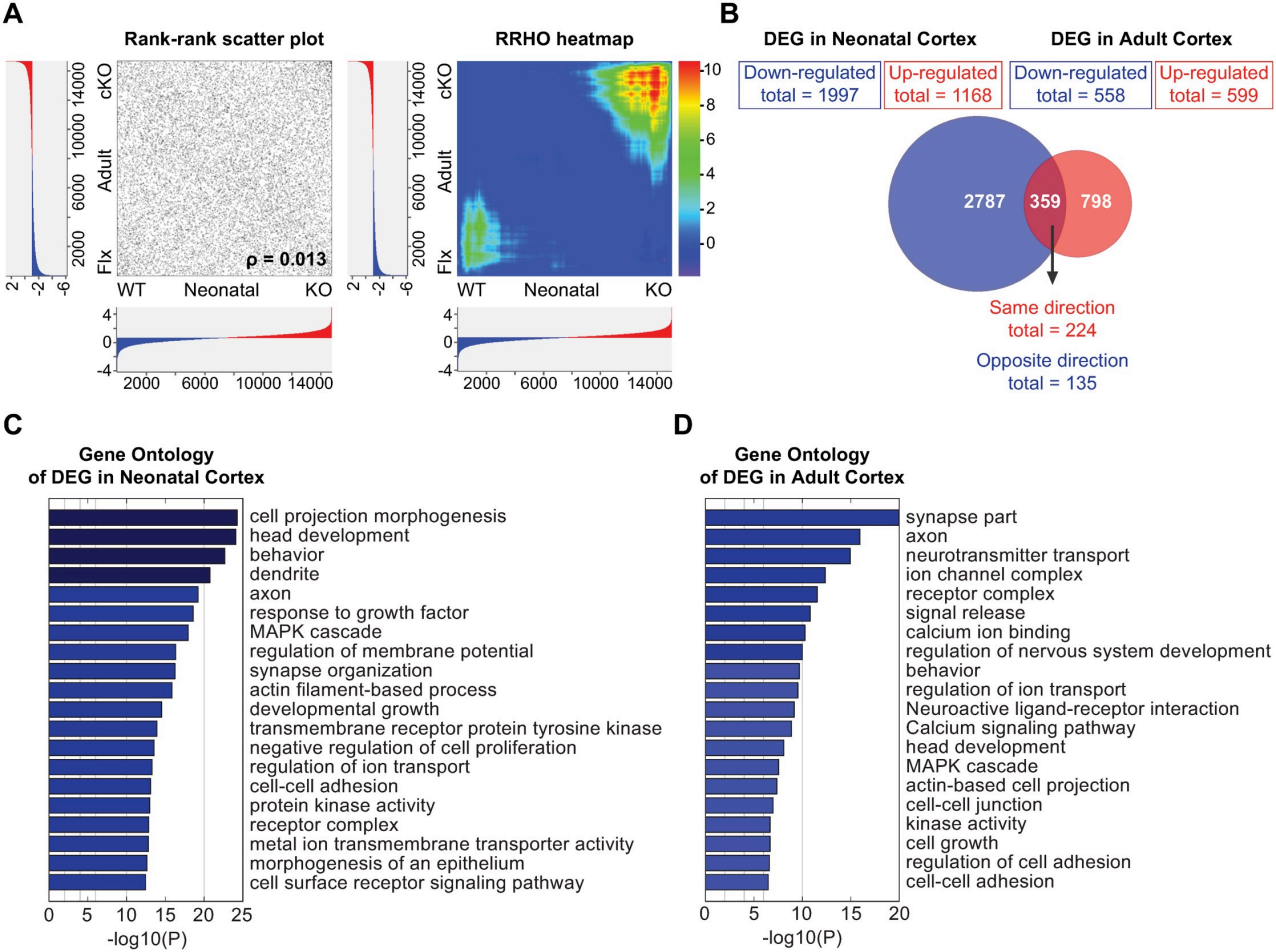
SATB2 exerts differential transcriptional effects in neonatal vs adult cortex. (A) Rank-rank scatter plot (left) and rank-rank hypergeometric overlap (RRHO) heatmap (right) comparing global gene expression signatures of SATB2-deficient and wild-type cortex at neonatal vs adult developmental stage. For each dataset (neonatal and adult), all expressed genes (gene counts more than 10) were ranked by their differential expression p-values and effect size direction based on SATB2 KO vs wild-type comparison. The significance of the overlap between the two gene lists is plotted as -log10 transformed hypergeometric test *p* values corrected for multiple testing by Benjamini and Yekutieli method. The range of the p-values is indicated in the color scale bar with negative values indicating under-enrichment. (B) Venn diagram illustrating the overlap between the differentially expressed genes (adjusted *p-*value < 0.05) in Satb2-deficient vs wild-type cortical tissue at P0 and adult stage (Fischer’s exact test, OR = 2.65, P < 0.0001; background, 19 626 protein-coding genes). (C) GO enrichment analysis of the differentially expressed genes between Satb2-deficient and wild-type neonatal cortex. (D) GO enrichment analysis of the differentially expressed genes between Satb2-deficient and wild-type adult cortex.

We next compared the two gene expression profiles using as a cutoff for differential expression adjusted p value < 0.05 (Fig 4B). We identified only 359 commonly regulated genes between SATB2-deficient and control cortices at adult vs neonatal stage (OR = 1.23, P = 0.0079). Notably, 135, i.e. approximately one third of the commonly regulated genes, were regulated in the opposite direction in the adult vs neonatal SATB2 mutant cortex, suggesting that a potential shift in the composition of SATB2-containing transcriptional complexes directly or indirectly determines whether transcription of these loci is repressed or activated. We next assessed the biological pathways influenced by SATB2-dependent transcription. GO analysis demonstrated enrichment of processes critical for cell projection morphogenesis and brain development in the genes regulated by SATB2 in the neonatal cortex (Fig 4C). By contrast, SATB2-dependent genes in the adult cortex were enriched in GO terms related to neuronal physiology and synapses including neurotransmitter transport, ion channel complex, calcium ion binding, neuroactive ligand-receptor interaction, calcium signaling pathway (Fig 4D). The GO terms enriched in the two SATB2-deficient transcriptomes are consistent with a differential role of SATB2 as a cell fate and neuron projection determinant at neonatal stage vs regulator of synaptic plasticity/physiology at the adult stage.

### Adult cortex SATB2 interactome is enriched for loss-of-function intolerant genes and genes associated with general cognitive ability

The human *SATB2* locus is highly constrained, rare mutations in the gene cause intellectual disability, and the gene influences cognitive ability in the general population [27–29]. Our transcriptome data showed that SATB2-dependent gene expression programs in the adult mouse cortex were enriched in GO terms associated with synaptic transmission and plasticity that are considered to underlie cognitive functions. We therefore asked if the genes encoding SATB2-containing protein complexes share the characteristics of *SATB2* and are associated with common variation in general cognitive ability.

**Fig 5:**
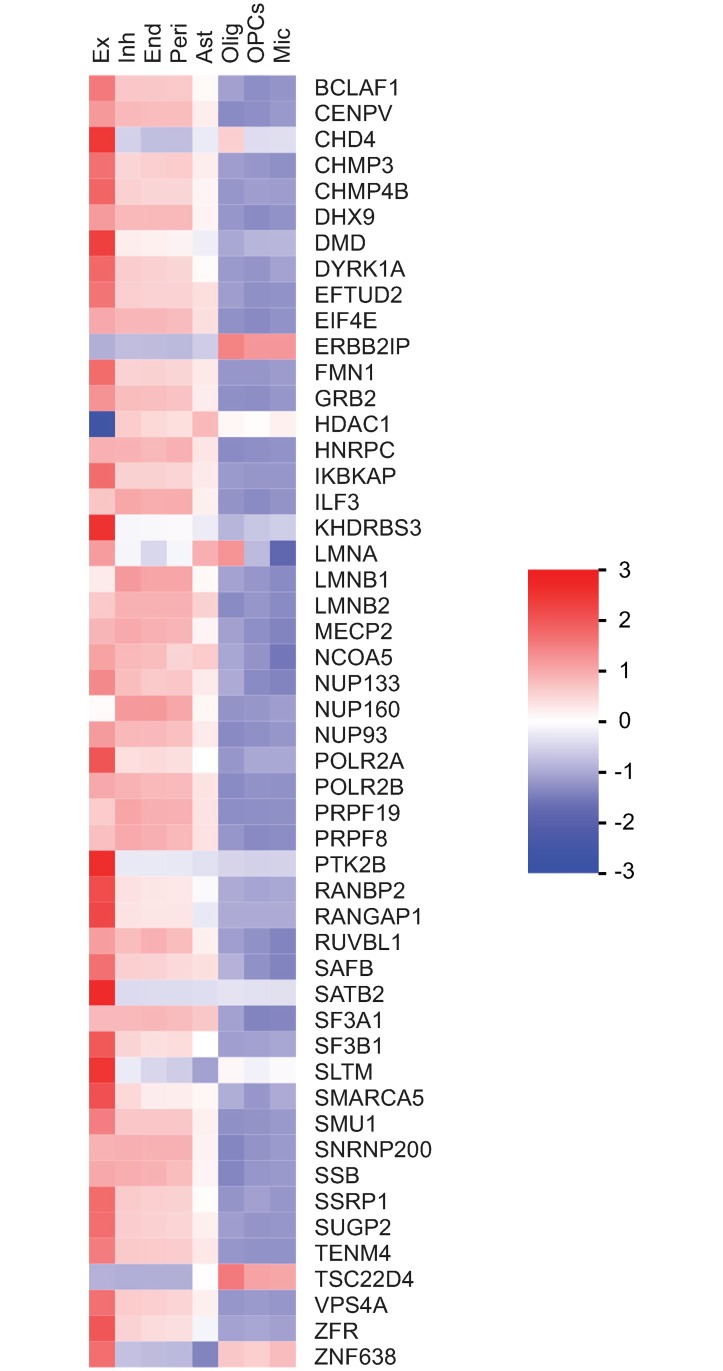
Expression of human orthologues of mouse SATB2 interactors across different cell types in adult human brain. Heatmap displaying the expression levels (Unique Molecular Identifier counts, [31]) of the human orthologs of mouse SATB2 interactors averaged across all cells within each cell type (4770 Excitatory neurons (Ex), 2337 Inhibitory neurons (Inh), 51 Endothelial cells (End), 45 Pericytes (Peri), 737 Astrocytes (Ast), 1758 Oligodendrocytes (Olig), 433 OPCs, 188 Microglia (Mic)). The color scale bar shows z-score values after z-score row normalization.

We first explored the expression of the human orthologs of mouse SATB2 interactors in human tissues using an expert-curated list of human-mouse homologous genes (http://www.informatics.jax.org/downloads/reports/index.html) and the available expression data in human tissues and brain cell types [30,31]. The human orthologs of mouse SATB2 interactors were found to be widely expressed in a broad spectrum of human tissues (S2 Fig). They are likely to be available for SATB2 interactions in human pyramidal neurons since they are co-expressed with SATB2 in the human adult cortex and excitatory pyramidal neurons (S3 Fig, Fig 5). Next, we tested the human SATB2 interactor gene-sets (adult and neonatal) for enrichment of highly constrained genes and intellectual disability (ID) genes. In the adult SATB2 interactome, 37 of 53 genes (69.8%) were loss-of-function (LoF) intolerant (S5 Table). In the neonatal SATB2 interactome, 28 of 40 genes (70%) were loss-of-function (LoF) intolerant (S5 Table). This identifies a very significant enrichment of highly constrained genes (adult, P = 5.05x10^-25^, neonatal, P = 6.71x10^-21^) and indicates that both SATB2 interactomes are under strong negative selection. Of the 53 genes in the adult SATB2 interactome, 6 (11.3%) are reported to be ID genes. In the neonatal SATB2 interactome, 6 of 40 genes (15%) are ID genes, representing a significant enrichment (P = 0.007) [32]. The respective ID genes and the intellectual disabilities reported in OMIM are listed in S5 Table.

To study the contribution of SATB2 interactomes to variation in cognitive function within the general human population, we employed data from genome-wide association studies (GWAS) of cognitive ability (CA) based on 269,867 individuals [33] and educational attainment (EA) based on 328,917 individuals [34]. Using MAGMA [35] to perform gene-set analysis (GSA) of the GWAS of CA and EA datasets, we found that the adult SATB2 interactome was significantly enriched for genes associated with CA (β = 0.337, P = 0.012) (S6 Table). We also observed of strong tendency for enrichment in the case of genes associated with EA (β = 0.201, P = 0.056). In contrast, common genetic variation in the neonatal SATB2 protein complexes was not significantly associated with common variation in either CA (β = -0.101, P = 0.728) or EA (β = 0.161, P = 0.169). Of note, 15 of the total of 91 genes encoding SATB2 interactors are reported to be contributing to CA and or EA based on single SNP and single gene analyses (S5 Table).

**Fig 6:**
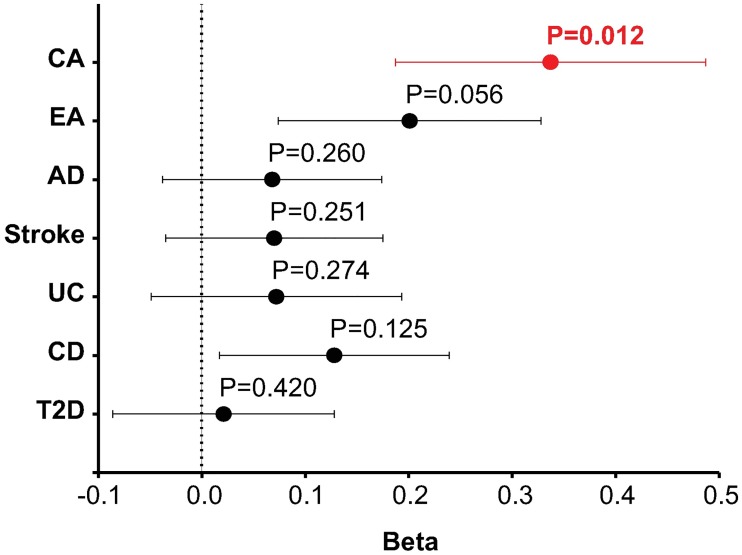
MAGMA GSA results for adult cortex SATB2 interactome using the summary statistics from multiple GWAS. GSA of SATB2 interactome in CA and EA plus post-hoc analysis of 2 brain-related disease and 3 non-brain diseases GWAS datasets. Phenotypes are listed on the y-axis (CA, Cognitive Ability; EA, Educational Attainment; AD, Alzheimer’s disease; UC, Ulcerative Colitis; CD, Cardiovascular Disease; T2D, Type II Diabetes). P-values are shown above each data point, which represent beta values (x-axis). Horizontal bars indicate error bars (SE). Data also supplied in S6 Table.

Brain-expressed genes are a major contributor to cognitive function [33]. It is possible that the enrichment detected here could be due to the SATB2 interactome representing a set of brain-expressed genes. However, the adult SATB2 interactome enrichments were robust to the inclusion in the analysis of both ‘brain-expressed’ (n = 14,243) and ‘brain-elevated’ (n = 1,424) gene-sets as covariates (P = 0.013 and P = 0.010, respectively; S7 Table). The SATB2 interactome is enriched for LoF intolerant genes and such genes are also enriched within genome-wide significant trait-associated loci [36]. To examine if the enrichments we detect for CA is a property of polygenic phenotypes in general, we obtained GWAS summary statistics for five phenotypes and we tested the adult SATB2 interactome for enrichment in each one. These were brain-related diseases (Alzheimer’s disease and Stroke) and non-brain-related diseases (Ulcerative Colitis, Cardiovascular Disease, and Type II Diabetes). Notably, the SATB2 interactome was not enriched for any of the five phenotypes tested (Fig 6; S6 Table). As *SATB2* is a risk locus for schizophrenia [15,16] and genes regulated by SATB2 contribute to schizophrenia [37], we also tested if the adult SATB2 interactome was enriched for genes associated with schizophrenia and other major neuropsychiatric disorders (Autism Spectrum Disorder, Attention-deficit/hyperactivity disorder (ADHD), Bipolar Disorder, and Major Depression Disorder) but no significant enrichments were detected (S7 Table).

## Discussion

Our comparative analyses of SATB2–dependent gene expression programs and SATB2 protein complexes in neonatal vs. adult mouse cortex provide insight into SATB2’s changing physiological brain functions. SATB2-dependent transcriptional responses in postmitotic forebrain neurons shift during brain maturation from regulation of cell fate and morphology to regulation of neurotransmission and plasticity. For SATB2-interacting protein complexes, we observe a complementary shift from transcriptional repression towards organization of higher-order chromatin structure. Analyses of the gene-sets derived from our proteomic experiments in human GWAS data also support a change in the function of SATB2 since only the genes encoding adult but not those encoding neonatal cortex SATB2 protein interactors contribute to human cognitive function.

Regarding SATB2 interaction partners, our data show that in both neonatal and adult mouse cortex SATB2 associates with proteins that form functionally interrelated protein networks—some shared, some unique to a single ontogenetic stage. Notably, the observed overlap between the adult and neonatal cortical data sets was found to be surprisingly limited. Even in the case of shared/similar protein complexes, the individual protein components appeared to be exchanged. Of note is the novel and unexpected interaction between SATB2 and the nuclear lamina, represented by LEMD2, LEMD3, LAP2 and BAF1 in the neonatal cortex, and Lamin A/C, Lamin B1, and Lamin B2 in the adult cortex. This finding provides a strong indication that tethering chromatin to nuclear lamina [38] is among the conserved molecular functions of SATB2. Other SATB2 interactors seem to be unique to the adult cortex, e.g. nuclear pore complex proteins, RNA helicases of the DDX family, some members of HNRNP family, RNA Pol II subunits, transcriptional repressors such as MECP2 and BCLAF1, the scaffold attachment factors SAFB and SAFA2. Another key finding of our proteomic analysis is that both adult and neonatal SATB2 interactomes are enriched in components of the molecular machinery involved in the *de novo* formation of stable chromatin loops [23], including RNA helicases of the DDX family and HNRNPs. As this type of chromatin-loop stabilizing machinery does not include classical regulators of chromatin architecture, such as cohesin and CCCTC-binding factor (CTCF) [39], our result suggests a role of SATB2-containing protein complexes in CTCF-independent stabilization of long-range chromatin contacts in cortical neurons. Given that regulation of 3D chromosomal conformations is likely to be of high relevance for memory formation and early adult-onset psychiatric diseases such as autism and schizophrenia [24,40], the datasets described here provide valuable information regarding candidate molecular regulators of spatial chromatin configuration linked to normal and impaired cognition.

The composition of the protein complexes SATB2 might be determined by expression levels, posttranslational modifications, or by alternative splicing of SATB2 and/or its interactors. We find that many of the adult cortex SATB2 interactors are expressed at lower levels in the adult cortex compared to neonatal cortex. Hence, it appears unlikely that their expression levels play a decisive role for SATB2 interaction. Furthermore, our immunoblotting data revealed indistinguishable molecular mass of SATB2 protein bands in neonatal and adult tissue lysates arguing against different isoforms of SATB2 being expressed at the two ontogenetic stages. Of note, SATB2 expression pattern shifts during brain maturation from superficial to all cortical layers ([3,6] and Allen Developing Mouse Brain Atlas, 2008). Accordingly, SATB2 in adult cortex is expressed in different subpopulations of cortical pyramidal neurons including both upper layer and deep layer excitatory neurons. This change in expression pattern might influence the composition of the available interaction partners.

As for the SATB2-dependent transcriptomes, we also discovered functional differences in the biological processes and pathways orchestrated by SATB2 as a transcriptional regulator at the two stages—from morphogenetic and differentiation processes in the neonatal cortex to mechanisms of neurotransmission and plasticity in the adult forebrain. Another difference is that the number of regulated genes is much higher at the neonatal stage compared to the adult. Although there is significant overlap in the gene sets affected by the loss of SATB2 in neonatal and adult brain, as could be expected, the number of commonly regulated gene is surprisingly small. Furthermore, we observed that a substantial number of SATB2-dependent genes were regulated in opposite directions between the two stages. Thus, SATB2 appears to have a modulatory function on the transcription of these genetic loci and by itself does not determine their repression or inhibition.

Our GSA provides further support for the divergence in the function of the neuronal SATB2 protein complexes between the two stages. Genes associated with cognitive function are enriched only in the adult but not in the neonatal SATB2 cortical interactome. The enrichment of the adult SATB2 interactome for common variants associated with cognition strongly indicates a role in human intelligence as already demonstrated for SATB2 itself [29,33,41]. Previous GSA using expert-curated pathways and GO gene-sets have identified dendrite and synapse-related pathways as significantly associated with major neuropsychiatric disorders [42,43] and cognitive function [33,41]. A connection of the pathophysiology of these diseases to postsynaptic complexes and a contribution to their common genetic architecture have been suggested [44,45]. More and more transcription factors and nuclear regulators are also emerging as genome-wide significant genes in the recent meta-analyses of GWAS of psychiatric diseases and human cognition [29,33,41,43,46], demonstrating the importance of the neuronal nucleus in addition to synapses for the pathophysiology of mental disorders. Yet, elucidating the contribution of specific nuclear processes to brain disorders by relying on curated canonical pathways and gene-sets has so far yielded unsatisfactory results. By contrast, the gene-sets in our experiments were derived from unbiased proteomic analyses, grouping genes solely based on their physical interaction with SATB2. This approach enabled us to discover unexpected associations. Most of the neocortical SATB2 interactors identified in our study are widely expressed across human non-neuronal tissues and also across different adult brain regions (S2 and S3 Figs). Although some of the corresponding genetic loci have been previously linked to neuropsychiatric diseases or cognition (S5 Table), the observed contribution of this combination of genes to general human cognitive ability, but not to other tested phenotypes is an unexpected finding. Now defined as a functional group, they present candidates for further studies on the mechanisms underlying intellectual disability and variability in intelligence. Moreover, by identifying SATB2 interactors as largely overlapping with the chromatin loop proteome [23], our results support the emerging concept that 3D chromatin architecture is a determinant of human cognitive ability.

## Material and methods

### Animals

Neonatal *Satb2* conditional mutants were generated by crossing *Satb2*^*flx/flx*^ mice [6] with *Nes-Cre* transgenic mice [47] on a C57BL/6 background. *Satb2*^*flx/flx*^::*Camk2a-Cre* mice have been described elsewhere [6].

### Antibodies

The following primary antibodies were used: anti-SATB2 (ab92446, Abcam); anti-SATB1 (ab92307, Abcam); anti-HDAC1 (10E2) (5356P, Cell signaling); anti-HNRNPL (ab156682, Abcam); anti-HNRNPL-like (4783S, Cell signaling); anti-HNRNPC1/C2 (M022726, BOSTER); anti-ZNF638 (orb215138, Biorbyt); anti-DHX9 (PA5-19542, Thermo Scientific); anti-CUX1 (M-222) (sc-13024, Santa Cruz); anti-Lamin A/C (N-18) (sc-6215, Santa Cruz); anti-ERK2 (C-14) (sc-154, Santa Cruz), anti-BANF1 (PU38143, a generous gift from T. Haraguchi [48]; anti-pan LAP2 (Lap2 2–12), a generous gift from R. Foisner [49], anti-CHMP3 (HPA015673, Atlas Antibodies).

### Immunoprecipitation, mass spectrometry and immunoblotting

Cortices were dissected from either neonatal or three month-old mice. Tissue was homogenized with a Dounce homogenizer in an IP lysis buffer (25 mM Tris/HCl pH 7.4, 150 mM NaCl, 1% NP-40, 1 mM EDTA, 5% glycerol; Pierce). The lysates were incubated for 10 min on ice with shaking, followed by a 15 min centrifugation at 13000 × g, 4°C. The supernatant was used in immunoprecipitation reactions using Dynabeads Protein G Immunoprecipitation Kit (Thermo Fischer) according to the manufacturer’s instructions. Briefly, 50 μl of protein G Dynabeads were covalently linked to 5 μg of anti-Satb2 antibody and incubated with cortical lysates overnight at 4°C. For MS sequencing, 1 mg of total protein was used as starting material. On the next day, the beads-antibody-protein complexes were washed 3 times with washing buffer, resuspended in 2 x Roti-Load sample buffer (Roth) for elution and incubated at 95°C for 5 min. The eluates were run on 6% SDS-PAGE gels.

Proteomic analysis was performed at the Protein Microanalysis Core Facility of Medical University of Innsbruck. Silver/Comassie-stained protein gels were divided into three molecular-weight ranges, cut and subjected to in-gel digestion as published previously [50]. Protein digests were analyzed using an UltiMate 3000 nano-HPLC system (Dionex) coupled to an LTQ Orbitrap XL mass spectrometer (Thermo Fischer) equipped with a nanospray ionization source. A homemade fritless fused silica microcapillary column (75 μm i.d. × 280 μm o.d.) packed with 10 cm of 3 μm reverse-phase C18 material (Reprosil) was used. The gradient (solvent A: 0.1% formic acid; solvent B: 0.1% formic acid in 85% acetonitrile) started at 4% B. The concentration of solvent B was increased linearly from 4% to 50% during 50 min and from 50% to 100% during 5 min. A flow-rate of 250 nl / min was applied. Protein identification was performed via Sequest, Proteome Discoverer (Version 1.3, Thermo Scientific) and the NCBInr database (*Mus musculus*) accepting variable modifications carbamidomethyl (C) and oxidation (M). Specific cleavage sites for trypsin (KR) were selected with two missed cleavage sites allowed. Peptide tolerance was ±10 p.p.m. and MS/MS tolerance was ±0.8 Da. The criteria for positive identification of peptides were Xcorr>2.3 for doubly charged ions, Xcorr>2.8 for triply charged ions, Xcorr>3.3 for four-fold and higher charged ions and a FDR of 0.01.

For validation of MS data, protein G Dynabeads were coated with 5 μg of anti-SATB2 antibody, the beads were mixed with 500 μg of cortical lysate and incubated overnight at 4°C. The antibody-protein complexes were eluted in 2 x Roti-Load sample buffer, separated by SDS-PAGE and immunoblotted with antibodies against the novel interacting partners. Immunobloting was performed as described previously [51]. Membranes were blocked with 5% milk powder in TBST (0.1% Tween 20 in TBS) for 1 h and then incubated overnight at 4°C with the corresponding primary antibodies diluted in blocking solution. After incubation with HRP-coupled secondary antibodies, the blots were developed using ECL reagent (GE Healthcare) and imaged with a FUSION-FX7 chemiluminescence detection system (Vilber Lourmat).

### RNA-seq analysis

RNA-seq analysis was carried out as previously described [6]. In brief, RNA was isolated from cortical tissue of 3 month-old *Satb2*^*flx/flx*^ and Satb2 cKO mice using Trizol (Thermo Fisher Scientific). Libraries were made according to Illumina standard protocols (TruSeq, Illumina) and sequenced as single-end reads on a HiSeq platform according to established procedures. RNA-seq reads were mapped to mouse reference genome (mm10) with STAR aligner [52]. Read counts were obtained using *featureCounts* [53] and normalized using the normalization algorithms of DESeq2 [54]. Differential gene expression analysis was performed with SARTools package [55]. A threshold cutoff of adjusted (Benjamini-Hochberg) p-value <0.05 was applied. RNA-seq data from McKenna et al. [17] (GSE68911) were re-analyzed using the same pipeline as for the adult cortex and the same threshold cutoff for differential expression was applied.

The gene expression profiles of SATB2-deficient vs wild-type cortices from the two datasets (neonatal vs adult stage) were compared by means of a rank-rank hypergeometric overlap (RRHO) analysis [26]. RRHO heat maps and rank scatter plot that graphically visualize correlations between two expression profiles were generated at http://systems.crump.ucla.edu/rankrank/.

### Functional annotation

Protein-protein interaction networks were extracted from the STRING 10.5 database (http://string-db.org/) and clustered by using k-means clustering method. “Experiments”, “Databases”, “Text-mining”, “Co-expression”, “Neighborhood”, “Gene Fusion”, and “Co-occurrence” were used as prediction methods with a medium confidence threshold (0.4).

Pathway and process enrichment analysis was carried out by using Metascape bioinformatics tool (http://metascape.org) [56] with the following ontology sources: KEGG Pathway, GO Biological Processes, GO Cellular Components, GO Molecular Functions and CORUM. All genes in the genome were used as the enrichment background. Terms with a p-value < 0.01, a minimum count of 3, and an enrichment factor > 1.5 (the ratio between the observed counts and the counts expected by chance) were collected and grouped into clusters based on their membership similarities. P-values were calculated based on the accumulative hypergeometric distribution, and q-values were calculated using the Benjamini-Hochberg procedure to account for multiple testing. Kappa scores were used as the similarity metric when performing hierarchical clustering on the enriched terms, and sub-trees with a similarity of > 0.3 were considered a cluster. The most statistically significant term within a cluster was chosen to represent the cluster.

ConsensusPathDB (http://cpdb.molgen.mpg.de/) was used to perform an overrepresentation analysis of the SATB2 interactomes using the complex-based sets (i.e. sets of genes whose protein products are members of the same annotated protein complex). The p-values were corrected for multiple testing using the FDR method.

### GWAS data and GSA

GWAS summary statistics were sourced for general CA [33], EA [34], Autism Spectrum Disorder [57], Attention-deficit/hyperactivity disorder (ADHD) [58], Bipolar disorder [46], Major Depression Disorder [43], Schizophrenia [16], Stroke [59], Alzheimer’s disease [60] Ulcerative Colitis [61], Type II Diabetes [62], and Cardiovascular Disease [63].

A GSA is a statistical method for simultaneously analyzing multiple genetic markers in order to determine their joint effect. We performed GSA using MAGMA (http://ctg.cncr.nl/software/magma) [35] and summary statistics from various GWAS identified above. MAGMA was chosen because it corrects for gene size and gene density (potential confounders) and has significantly more power than other GSA tools [64]. An analysis involved three steps. First, in the annotation step we mapped SNPs with available GWAS results on to genes (GRCh37/hg19 start-stop coordinates +/-20kb). Second, in the gene analysis step we computed gene P values for each GWAS dataset. This gene analysis is based on a multiple linear principal components regression model that accounts for linkage disequilibrium (LD) between SNPs. The European panel of the 1000 Genomes data was used as a reference panel for LD. Third, a competitive GSA based on the gene P values, also using a regression structure, was used to test if the genes in a gene-set were more strongly associated with either phenotype than other genes in the genome. Sets of ‘brain-expressed’ (n = 14,243 genes) and ‘brain-elevated’, i.e. genes that show an elevated expression in brain compared to other tissue types (n = 1,424) gene-sets were sourced from the Human Protein Atlas (https://www.proteinatlas.org/humanproteome/brain) and used as covariates in a conditional MAGMA GSA.

### Enrichment analysis

Genes were categorized as LoF intolerant if their probability of being LoF intolerant (pLI) metric was ≥ 0.9 based on the analysis of exome data for 60,706 humans by the Exome Aggregate Consortium [36]. A list of primary ID genes (n = 1,069) was sourced from the curated SysID database of ID genes (http://sysid.cmbi.umcn.nl/) [32]. A list of proteins recruited to the stabilized chromatin loops was sourced from [23]. Enrichment analysis of these gene/protein lists with our gene-sets was performed using 2x2 contingency tables with genes restricted to those annotated as protein coding using a background set of 19,626 genes (https://www.ncbi.nlm.nih.gov/).

## Supporting information

S1 Fig Functional enrichment analysis of unfiltered SATB2 interactomes. Metascape enrichment analysis of neonatal (A) and adult cortex (B) SATB2 interactomes, identified without applying the “nuclear localization” filter. (C) Venn diagram depicting the overlap between unfiltered neonatal and adult cortex SATB2 interactomes (Fischer’s exact test, OR = 13.24, P = 0.0114 (including SATB2 itself), OR = 6.61, P = 0.1448 (excluding SATB2 itself); background, 19 626 protein-coding genes). OR, odds ratio.
(TIF)

S2 Fig Expression of human homologs of mouse S2 interactors across different human tissues. Data from the GTEx Consortium (dbGaP Study Accession: phs000424.v2.p1, [30]) were used to generate the heatmaps. Expression values are presented as median transcripts per million by tissue. The color scale bar shows z-score values after z-score row normalization.
(TIF)

S3 Fig Expression of human homologs of mouse SATB2 interactors across different human adult brain regions. Data from the GTEx Consortium (dbGaP Study Accession: phs000424.v2.p1) [30] were used to generate the heatmaps. Expression values are presented as median transcripts per million by brain region. The color scale bar shows z-score values after z-score row normalization.
(TIF)

S1 Table List of proteins identified as interactors of SATB2 in the neonatal cortex by co-IP /MS analysis. Shown are percent coverage (calculated by dividing the number of amino acids in all found peptides by the total number of amino acids), the number of distinct peptide sequences in the protein group and the protein score (calculated as the sum of the scores of the individual peptides) for each individual interaction partner. *, HDAC1 was represented by only one peptide. We included it in the list because SATB2-HDAC1 interaction has been described in the literature and we validated it by independent IP of endogenous SATB2 from cortical lysates followed by IB (Fig 2).
(XLSX)

S2 Table List of proteins identified as interactors of SATB2 in the adult cortex by co-IP /MS analysis. Shown are percent coverage (calculated by dividing the number of amino acids in all found peptides by the total number of amino acids), the number of distinct peptide sequences in the protein group and the protein score (calculated as the sum of the scores of the individual peptides) for each individual interaction partner. *, HDAC1 was represented by only one peptide; CHMP3 was identified in only one replicate. We included them in the list because we were able to validate the interaction with SATB2 by independent co-IP/IB experiments (Fig 2).
(XLSX)

S3 Table ConsensusPathDB over-representation analysis for the neonatal SATB2 interactome. (XLSX)

S4 Table ConsensusPathDB over-representation analysis for the adult SATB2 interactome. (XLSX)

S5 Table Status of the genes encoding SATB2-interacting proteins in relation to LoF intolerance, intellectual disability, cognitive ability, educational attainment, neuropsychiatric phenotypes, and other human diseases or traits. (XLSX)

S6 Table MAGMA gene-set analyses of the SATB2 interactomes in cognitive function as opposed to other polygenic diseases. (XLSX)

S7 Table MAGMA gene-set analyses of the adult cortex SATB2 interactome in neuropsychiatric disorders. (XLSX)

S8 Table List of proteins identified as interactors of SATB2 in the neonatal cortex by co-IP /MS analysis without applying the “nuclear localization” filter. (XLSX)

S9 Table List of proteins identified as interactors of SATB2 in the adult cortex by co-IP /MS analysis without applying the “nuclear localization” filter. (XLSX)
