# Supplementary figures and images for: Genes encoding SATB2-interacting proteins in adult cerebral cortex contribute to human cognitive ability
Source: PLoS Genet. 2019 Feb 6;15(2):e1007890. doi: 10.1371/journal.pgen.1007890 (PMC6364870; doi:10.1371/journal.pgen.1007890)

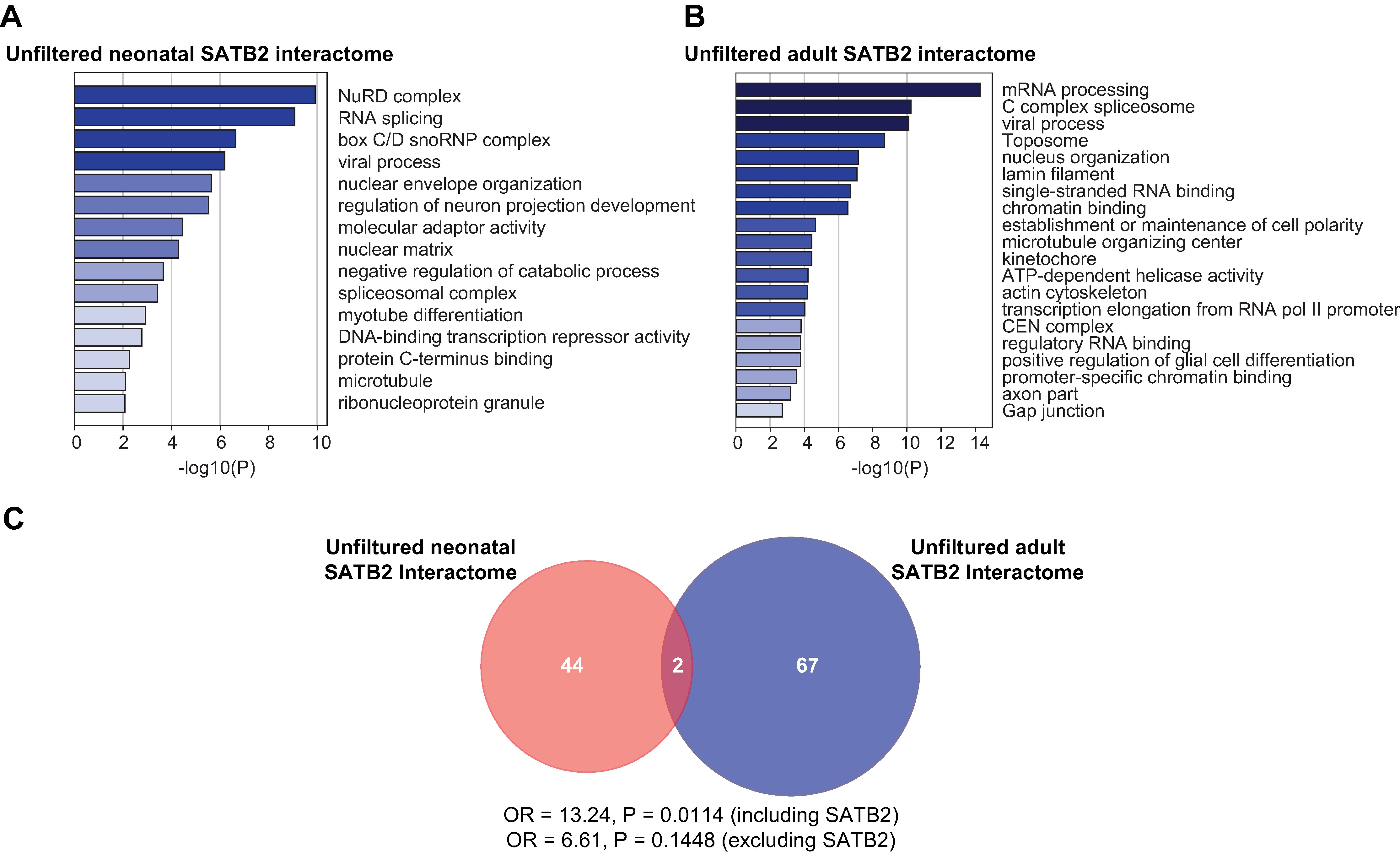

Supplement: S1 Fig — Metascape enrichment analysis of neonatal (A) and adult cortex (B) SATB2 interactomes, identified without applying the “nuclear localization” filter. (C) Venn diagram depicting the overlap between unfiltered neonatal and adult cortex SATB2 interactomes (Fischer’s exact test, OR = 13.24, P = 0.0114 (including SATB2 itself), OR = 6.61, P = 0.1448 (excluding SATB2 itself); background, 19 626 protein-coding genes). OR, odds ratio. (TIF) [file pgen.1007890.s001.tif]

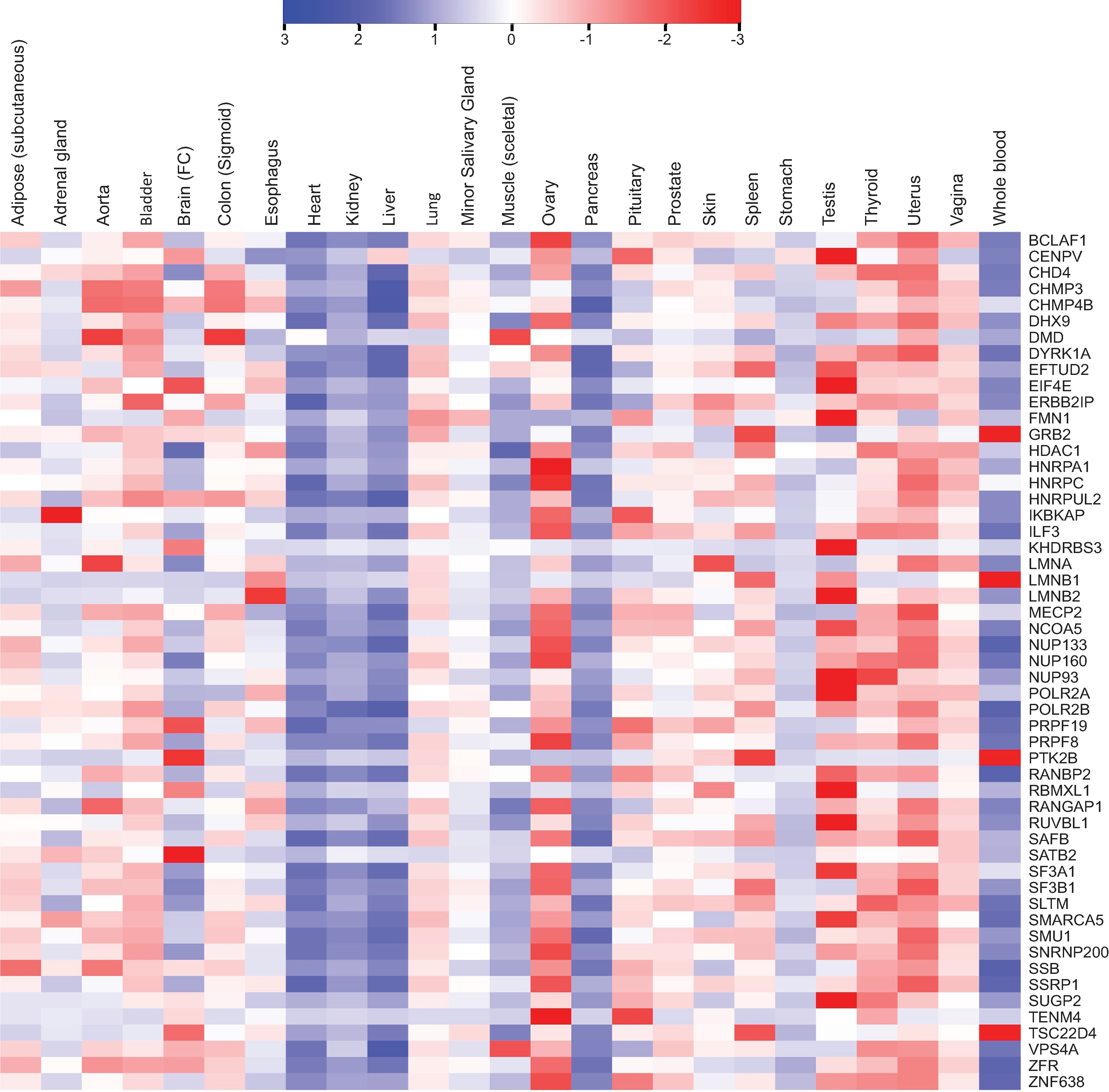

Supplement: S2 Fig — Data from the GTEx Consortium (dbGaP Study Accession: phs000424.v2.p1, [30]) were used to generate the heatmaps. Expression values are presented as median transcripts per million by tissue. The color scale bar shows z-score values after z-score row normalization. (TIF) [file pgen.1007890.s002.tif]

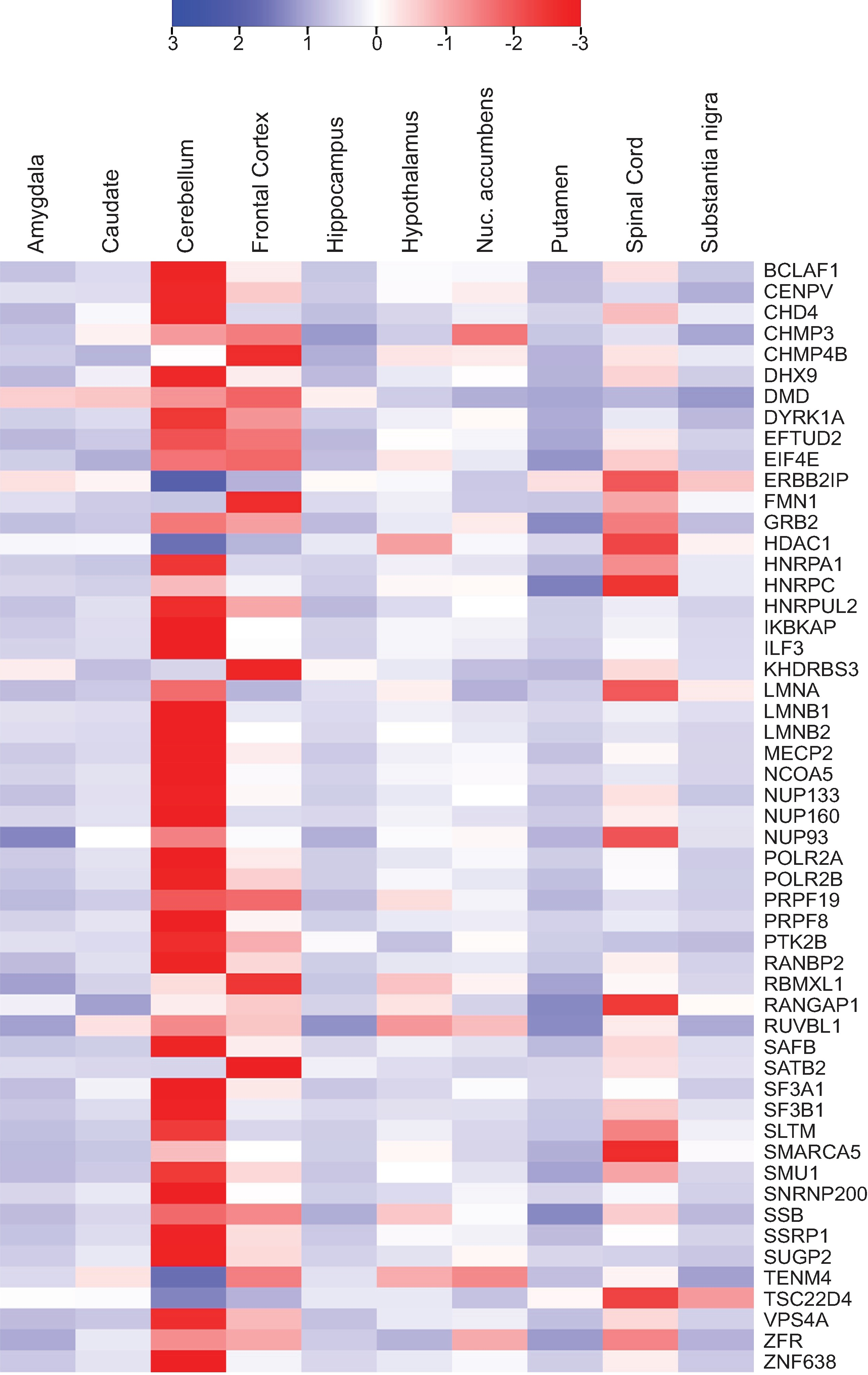

Supplement: S3 Fig — Data from the GTEx Consortium (dbGaP Study Accession: phs000424.v2.p1) [30] were used to generate the heatmaps. Expression values are presented as median transcripts per million by brain region. The color scale bar shows z-score values after z-score row normalization. (TIF) [file pgen.1007890.s003.tif]
